# Promoting sport participation during early parenthood: a randomized controlled trial protocol

**DOI:** 10.1186/s13063-020-4158-x

**Published:** 2020-02-27

**Authors:** Stina J. Grant, Mark R. Beauchamp, Chris M. Blanchard, Valerie Carson, Ryan E. Rhodes

**Affiliations:** 1 Behavioural Medicine Laboratory, Faculty of Education, University of Victoria, PO Box 3010, STN CSC, Victoria, BC V8W 3N4 Canada; 2 University of British Columbia, Vancouver, BC Canada; 3 Dalhousie University, Halifax, NS Canada; 4 University of Alberta, Edmonton, AB Canada

**Keywords:** Sport participation, Parents, Well-being, Psychosocial health

## Abstract

**Background:**

Adult participation in sport is associated with important positive psychosocial outcomes. Despite the multitude of benefits that have been linked to sport participation, adult participation rates in Canada remain low. Parents with young children represent a demographic that may benefit considerably from sport participation, given the prevalence of inactivity coupled with increased levels of psychosocial distress among this group. This study aims to evaluate the efficacy of two types of sport participation (individual sport and team sport) on key psychosocial outcomes compared with a “personal time” control condition among parents with young children.

**Methods/design:**

The three-arm, parallel design, single-blind, randomized controlled trial will compare a team sport condition, an individual sport condition, and a “personal time” control condition over 3 months. Parents are eligible if they have a child under 13 years of age, are not participating in a sport at baseline, and are not meeting Canadian Physical Activity Guidelines. Psychosocial variables (quality of life, relationship satisfaction, social functioning, parental stress, and enjoyment) will be assessed at baseline, 6 weeks, and 3 months. A total of 161 parents have been recruited thus far from the Greater Victoria region in British Columbia, Canada. The study is ongoing with a target goal of 240 participants and an anticipated completion date of December 2021.

**Discussion:**

This protocol describes the implementation of a randomized controlled trial that evaluates the effectiveness of sport participation for increasing positive psychosocial outcomes. This information could prove useful for future adult sport participation and potentially inform public health initiatives involving parents and families.

**Trial registration:**

ClinicalTrials.gov, NCT02898285. Registered 13 September 2016.

## Background

Recreational sport participation among adults is associated with a multitude of positive psychological and social health outcomes, including overall psychological well-being/life satisfaction, lower stress, higher social functioning, greater vitality, enjoyment, and a sense of community belonging when compared with adults who do not participate in sports [1]. This plethora of beneficial outcomes are well aligned with the key psychosocial health objectives of the Canadian Sport Policy [2]. Notably, team sport participation has been associated with even higher levels of these beneficial outcomes when compared with adults who participated in individual sports [3], an added benefit proposedly due to the social nature of team sports [1, 4]. Sport participation may also serve the dual aim of increasing physical activity (PA) to reap the well-established physical and psychological benefits associated with regular PA, such as reduced risk for chronic disease and premature mortality, reduced depression and anxiety, and improved overall quality of life [5].

Despite the array of positive psychosocial outcomes emerging from early-phase research on sport participation, few adults participate in sports. Indeed, participation rates among Canadians remain alarmingly low, with convincing evidence that sport participation declines steeply from late adolescence (54% participation) to adulthood (23% participation) [6]. To contextualize this finding, adult participation rates are much lower than in countries with similar climates, such as those in Scandinavia (42% participation) [7, 8], as well as countries in northern Europe (40%) [8], and compared with countries with similar population/economic distribution, such as Australia (56%) [9, 10]. Unsurprisingly, improving sport participation is a priority of the Canadian Sport Policy [2] and continues to be a critical issue in Canada [11].

When considering low participation rates among adults and potential psychosocial benefits, some groups may have a greater possibility to benefit. One such subset may be parents with young children. Given the demands of parenthood, parents of young children often report higher levels of psychosocial distress, such as increased depressive symptoms, lower vitality, and social isolation compared with age-matched adults without children [12–14]. Importantly, parents also report a significant decline in overall PA, inclusive of sport participation [15–17]. To this end, in a systematic review examining PA in studies with longitudinal designs, parenthood emerged as a significant predictor of PA decline [18]. This PA decline among parents is of particular concern because of the ensuing unlikelihood of meeting the public health guidelines recommended to optimize benefits [19]. Clearly, parents represent an important sport promotion demographic that may stand to gain considerable psychological, social, and health benefits.

To date, there has been no experimental investigation into the effects of sport participation on key psychosocial outcomes among parents. Furthermore, the effects of an individual sport versus a team sport have not been investigated empirically in the context of parents. This is a noteworthy limitation of prior research, given the promising effects of sport participation on psychological and social health [1]. Indeed, Eime and colleagues [1] recommended further investigation into the causal link between sport participation and psychosocial outcomes. To our knowledge, this study will be the first randomized controlled trial to examine this relationship. Therefore, the purpose of this paper is to describe the protocol of a study aiming to evaluate the efficacy of two types of sport participation (individual sport and team sport) regarding key psychosocial outcomes compared with a “personal time” control condition (a suitable comparator because it is time spent away from children that is not active in nature) among parents with young children.

## Methods/design

### Theoretical framework

Although the primary aim of the study is focused on sport participation outcomes, a secondary objective is to understand the mechanisms involved in sport participation behavior among parents. Theoretical frameworks act as useful guides for understanding behavior. Although many theories have been applied to understand sport participation, the Sport Commitment Model of Scanlan and colleagues [20] offers a suitable framework because it was specifically designed to examine reasons why individuals continue their participation in sport.

According to the model, commitment (i.e., desire and resolve to continue) is the primary mediator of sport participation. Because the model was built on hedonic theory [21] and intrinsic motivation [22], enjoyment (pleasure, fun, liking of sport) is thought to be the primary motive behind participation. There are four additional antecedents that are considered important to sport commitment: involvement alternatives (the attractiveness of the most preferred alternative), personal investment (personal resources put into the activity), social constraints (expectations or norms that create feelings of obligation), and involvement opportunities (valued opportunities present only through continued involvement).

The model has sound validation in various sport domains, including amateur youth sport, elite-level sport, health club fitness participation, and competitive sport among adolescents [20, 23–25]. Studies show that all five of the proposed antecedents of the model can predict commitment and that commitment reliably predicts sport participation. However, there is a paucity of research applying the model in a recreational sport setting among adults. To this end, only one study has applied the model to understand adult recreational sport [26]. This study sought to validate the sport commitment model in an adult recreational setting, namely tennis. Acceptable indices of fit indicated that the model has utility in understanding adult recreational sport participation. Furthermore, the model seems particularly applicable to parents because relevant antecedents of participatory behavior such as alternatives (child care and support, domestic activities), involvement opportunities (friendship and social interaction), and social expectations may play key roles in defining who continues to participate in sports. Finally, the model may assist in identifying potential mechanisms for the psychosocial outcomes. The present study aims to examine the conjecture that enjoyment and opportunities for social involvement act as key mediators for team sport participation over individual sport involvement [27, 28]. In sum, the present study seeks to fill a gap in the contemporary literature by examining the effects of both individual and team sport compared with a control condition, as well as the social and enjoyment mechanisms proposed as mediators of this relationship through application of the Sport Commitment Model [20].

### Pilot research

This research builds on both the extant literature and several pilot projects. First, a systematic review by Eime and colleagues [1] on sport participation and psychosocial outcomes provides valuable data from which the rationale for the experimental design was drawn. In addition, a community survey of 76 inactive parents residing in Greater Victoria, British Columbia, Canada, with children under the age of 13 years was conducted in order to explore the types of sports in which parents would have the greatest interest. Responses for the survey aligned with Canadian adult sport preferences [6]: The majority of parents chose soccer, basketball, ice hockey, dragon boating, and volleyball as preferred team sports, whereas the most preferred individual sport programs included running, swimming, and cycling. Finally, an environmental scan was conducted that investigated all of the adult recreational team sport and individual sport programs in Greater Victoria as well as adjacent licensed childcare opportunities. The scan established that all of the preferred sports from the survey had leagues/programs that run in every season. The well-matched and accessible sport and childcare opportunities indicated the plausibility of sport participation required for this study.

### Present research aims and hypotheses

The primary research question is whether a team sport condition increases positive psychosocial outcomes (quality of life, relationship satisfaction, social functioning, perceived parental stress, enjoyment) compared with an individual sport condition and a “personal time” control condition involving a choice-based pastime spent away from children. We hypothesize that the team sport condition will result in significantly greater changes in psychosocial outcomes than the other two conditions at the primary endpoint (3 months). Furthermore, the individual sport condition will also yield significantly larger changes in psychosocial outcomes than the personal time condition after 3 months of participation. Three additional secondary research questions will also be examined:

1. Can participation in the team sports and individual sports conditions be explained by the constructs of the Sport Commitment Model?
  We hypothesize that participation will be predicted by sports commitment as per the tenets of the Sport Commitment Model. Commitment will be predicted primarily by enjoyment (+), social constraints from family obligations/involvement alternatives (−), and social involvement opportunities/personal investments (+).
2. Can group differences among these motivational and behavioral outcomes be explained through a mediation model?
  We propose that the covariance of the assigned conditions on psychosocial outcomes will be explained by sport participation. In turn, the covariance between participation and assigned conditions will be explained by salient underlying motives from the Sport Commitment Model. In particular, enjoyment will explain the differences between both sport conditions, but the better psychosocial outcomes from team sports will be explained by the additional involvement opportunities of friendship and social interaction.
3. Is there a seasonal, gender, dual/single parent, age of child, or type of sport difference across primary outcomes by assigned condition?
  Differences in season, gender, age of child, and type of sport are exploratory research questions. It is possible that both sport conditions may have participation lowered by weather conditions in the winter. Men may participate in sport more due to lower childrearing expectations; however, there is currently limited research to support this conjecture. Similarly, younger children may impact sport participation because of the considerable care needs of young children. Finally, it is possible types of sport could differ in their psychosocial outcomes for reasons such as the structure or environment of participation.

### Design

**Fig. 1:**
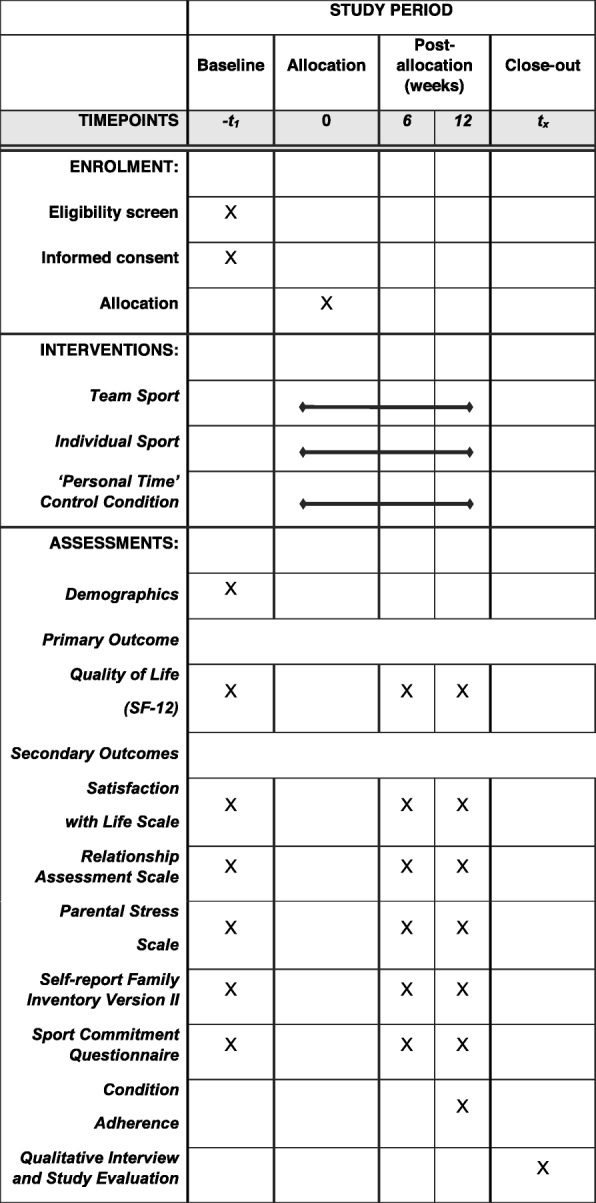
Schedule of enrollment, conditions, and assessments according to Standard Protocol Items: Recommendations for Interventional Trials (SPIRIT) diagram

This study is a three-arm, parallel design, single-blind, randomized controlled trial. Written informed consent is obtained from participants before study enrollment. Participants are then assigned to one of three groups for a duration of 3 months: team sport choice condition, individual sport choice condition, or personal time control condition. Primary outcome measures in all three groups are assessed at baseline, 6 weeks, and 3 months. The design, conduct, and reporting of the trial has and continues to follow Standard Protocol Items: Recommendations for Interventional Trials (SPIRIT) guidelines [29] and will conform with Consolidated Standards of Reporting Trials (CONSORT) guidelines [30]. A SPIRIT checklist is provided as an Additional file and the SPIRIT diagram is included as Fig. 1. The randomized controlled trial is registered with the clinical trials registry maintained by the National Library of Medicine at the National Institutes of Health (ClinicalTrials.gov identifier NCT02898285).

### Participants and eligibility

Parents are considered eligible if they reside in Victoria, British Columbia, and have at least one child under 13 years of age who resides in the home. This cutoff was delimited as such because it represents the lower bound of the teenage years, when children still have substantial care needs. It follows parents with children under 13 have numerous familial obligations and subsequently experience notable time constraints. Both mothers and fathers are eligible, inclusive of those with single status. Parents must not engage in regular sport participation (operationally defined as not having participated in an organized sport in the month prior to baseline). Parents must also be below the recommended physical activity guidelines (150 min of weekly moderate to vigorous physical activity [19]), which is determined via self-report at the initial screening interview. Parents need to be willing to participate in a team sport in addition to being deemed safe to engage in moderate-intensity physical activity (assessed via the Get Active Questionnaire [31];). Those individuals who are not ready or able to participate in moderate-intensity physical activity will be excluded for safety reasons. Mental health conditions such as depression and anxiety do not preclude participation, because these are not contraindications to beginning a physical activity program [32].

### Recruitment procedure

Parents are recruited primarily through advertisements on online interest sites and social media platforms. Specifically, posts targeted for a local parent audience are advertised weekly on Facebook and Instagram. Additionally, print advertisements are placed at recreation centers, health care centers, children’s recreation classes, and coffee shops every few months. Pamphlets are also offered at biweekly booths at local community markets and family-oriented events. To ensure diversity of the study population, the city was systematically stratified into regions, and facilities from each region were randomly selected and contacted for recruitment as per a previously refined recruitment strategy. Recruitment also takes place on a referral basis whereby current participants are invited to pass study information to others. Finally, incentives for participation include honoraria subsidizing the cost of the activity (up to a total of $80) as well as assistance with childcare costs if needed (up to $25 weekly).

Recruitment is ongoing across the length of the study. Because enrollment takes place in waves that align with seasons for adult sport leagues/programs, those who demonstrate interest are placed on a wait-list to enroll during the uptake for the next wave.

Those interested in taking part are invited to contact the researchers via email or phone. If interest in the study is expressed, potential participants are sent additional study details by email. At the next stage of the enrollment process, potential participants are formally screened over the phone with a recruitment officer or the project coordinator. If they meet the eligibility criteria, they are booked for a baseline assessment or alternatively placed on a wait-list, depending on the timing of the next wave.

### Randomization, allocation, and blinding

Randomization is performed by the project coordinator using Excel Sheet Randomization at a 1:1:1 allocation ratio. A research assistant then prepackages envelopes containing certificates that indicate condition assignment along with a list of potential ideas and resources for that condition. A second research assistant who is blind to participants’ condition presents the participants with their randomized allocation.

### Outcome measures

#### Primary outcome measures

##### Change from baseline to 3 months in parental psychosocial outcomes

Assessments of psychosocial outcomes in all three groups are collected through online questionnaires (available on SurveyMonkey) and occur at three time points: baseline, 6 weeks, and 3 months.

##### Quality of life

Psychosocial outcomes are primarily assessed using the 12-item Short Form Health Survey (SF-12) [33, 34], which measures health-related quality of life on a range of functional domains, including vitality, social functioning, and overall well-being. The domains assessed in the SF-12 have prior application in the sport domain [27, 35]. The SF-12 has been validated for adult populations, and there is established evidence for the reliability of scores derived from the instrument [34].

#### Secondary outcome measures

##### Psychosocial outcomes and psychosocial distress

Additional quality of life outcomes are assessed via the Satisfaction With Life Scale, which has established reliable and valid test scores [36] and has been used in prior sport research [37]. Given our parent sample, we also measure relationship satisfaction with the Relationship Assessment Scale, which has shown adequate validity and reliability [38, 39], and parental stress with the previously validated Parental Stress Scale, which has shown a high level of internal consistency [40]. Finally, family functioning is assessed using the self-report Family Inventory Version II, which measures familial health/competence, conflict, cohesion, leadership, and emotional expressiveness [41].

##### Sport commitment and sport participation

Parents are also assessed on the constructs of the Sport Commitment Model [20] at three time points: baseline, 6 weeks, and 3 months. The Sport Commitment Model [20] is measured by the associated sport commitment questionnaire with the adjusted measures for adult samples relevant to parents of children [26, 42]. Previous sport commitment research among adult fitness participants has shown the questionnaire to have acceptable validity and reliability in the measurement of sport commitment, enjoyment, involvement alternatives, personal investments, social constraints, and involvement opportunities [24]. The questionnaire wording was slightly modified in order to be framed for both sport and physical activity.

Additionally, at the final 3-month measurement, condition adherence/sport participation is assessed with a study-created item, “How was your attendance for your condition (team sport, individual sport, or personal time)?” with possible answers of “did not adhere at all,” “missed over half the sessions (at least 6),” “missed 3–4,” “missed 1–2,” and “did not miss any sessions.” An additional item assesses reasons for missing sessions with responses including common barriers for parents, such as “too tired,” “couldn’t get childcare,” “work commitments,” and “family commitments.” Last, four items rated on a scale from 1 (strongly disagree) to 5 (strongly agree) asks participants the extent to which their condition was enjoyable, beneficial, or challenging and how much they looked forward to their weekly activity. Condition adherence/sport participation is also complemented by a phone-assisted report of attendance at the 6-week assessment and an in-person report at the final 3-month interview.

##### Demographics

A brief section in the baseline questionnaire is used to assess characteristics, including age, gender, marital status, ethnicity, level of education, health background, employment information, sleep, smoking, alcohol, and eating behavior. Child-related demographic information is also collected, including the number of children, age of children, and gender of children. Importantly, items related to childcare are measured at all time points in order to assess whether participants have a formal or informal childcare provider, average hours of childcare daily, and difficulty finding childcare. Any changes in health are also assessed at all measurement points. Last, a commute section asks the total length of time (one way) it takes to get to the allocated choice activity, as well as the mode of transportation used to travel there.

##### Study evaluation

A brief end-of-program interview is conducted with two main aims: to gain a deeper understanding of parents’ attitudes and beliefs surrounding the importance of personal time or sport and to seek richer and in-depth information regarding their experiences with the program. Providing an opportunity to expand information verbally serves these aims and allows the program outcomes to be better explained. This is accomplished through conducting a 10-minute audio-recorded interview with a member of the research team. The interview questions touch on benefits, types of activities, changes in well-being, enjoyment, and barriers or the potential removal of barriers. Participants are then invited to provide any additional thoughts or suggestions on how the program might be improved. Last, the interviewer asks participants whether they will continue with their allocated condition after the study.

### Data management and confidentiality

Confidentiality procedures are outlined in the consent form (*see* Additional file 2) and explained during informed consent procedures conducted by the research assistant at the baseline assessment. In short, each participant is provided with an identification number. Hard copies of any documentation are kept in a locked and secure environment (locked laboratory and cabinets) at the University of Victoria. Any data or personal information stored on computers is kept on a secure server. Questionnaire data are stored on SurveyMonkey servers in Canada.

### Analysis strategy

Missing data will be inspected to determine the appropriate imputation procedures [43], and normality of all variables will be checked to determine whether any transformations are required. To determine whether psychosocial outcomes change over time similarly for all three conditions (i.e., to address objective 1), a level 1 model will be specified wherein the intercept (i.e., value of the outcome at baseline) will be allowed to vary randomly (i.e., vary across participants) and the slope for the linear trend will be constrained to be fixed (i.e., the same across participants), controlling for any significant covariates at level 2. Additionally, dummy variables will be created for condition (team sports: 1 = yes or 2 = no; individual sport: 1 = yes or 2 = no; control: 1 = yes or 2 = no) at level 2, with the team sport and individual sport variables being added to the model to predict the intercept and slope at level 1. In doing so, the control group is compared with the other two groups to determine if baseline psychosocial outcomes are similar across conditions and whether the change is similar across conditions. Follow-up analyses will be conducted for the group comparison. To determine whether participation within the team and individual sport conditions is explained by the Sport Commitment Model constructs (i.e., secondary objective 1), a time-varying covariate analysis will be used. For example, within the team sport condition, participation will be predicted by a level 1 model that includes a random intercept, fixed slope, and the time-varying Sport Commitment Model constructs. The same will then be done for the individual sport condition. To determine whether the change in the underlying motives explains the potential change in sport participation during the intervention similarly for all three groups (i.e., to address secondary objective 2), a time-varying covariate mediation analysis approach will be used following the procedure outlined by Krull and MacKinnon [44] for level 1 mediation. Briefly, the analyses needed to establish mediation will treat the underlying motives as time-varying covariates at level 1 of the model. Then, the dummy-coded condition variables will be entered at level 2 to determine if the mediation relationships are similar across groups. Finally, to determine whether there is a seasonal, gender, or sport type (within team or individual) difference across the primary and secondary outcomes (i.e., to address secondary objective 3), each variable will be entered into the various models at level 2 to predict the intercepts and slopes at level 1. Doing so will determine if they impact the change in the various outcomes across time.

### Justification of sample size

Given the hierarchical nature of the data (i.e., the three measurement occasions at level 1 were considered to be nested within the participant at level 2), the optimal design program for power estimation of hierarchical linear models [45] was used. With three measurement occasions, three groups, and a small to moderate effect size (0.40), a total of 207 participants (i.e., 67 parents per condition) were needed to show significant differences over time. Inclusive of a potential 25% attrition rate, the total sample size required is 240.

### Intervention description, schedule, procedures, and protocol

After interested participants are deemed eligible and enrolled, they are booked for a baseline meeting. At this meeting, informed consent is obtained by a research assistant, then participants are asked to complete a baseline questionnaire containing demographic, sports commitment/behavior, and quality of life items (*see* Fig. 1 for enrollment schedule). Next, participants are randomized to one of three conditions. A research assistant who is blind to each participant’s condition presents the participant with a prepackaged envelope that reveals the condition allocation. The research assistant then outlines possible activity options and provides resources to facilitate registration in an activity program when required. Condition allocation will not require alteration of usual care pathways (including use of any medication), and these will continue for all trial arms.

Participants in the control condition are asked to use the honorarium funds to treat themselves to a weekly “night out” or “personal time” of their choice, as long as the time is not physically active and the time is spent away from their children. Examples of personal time are provided, such as going for coffee, going to the movies, or taking a workshop or class. Participants in the personal time control condition are told they will not receive any further contact until the next assessment period.

Participants in the team sport choice condition are asked to make a selection from a customized and up-to-date handout of the environmental scan of the available adult team sport programs in Greater Victoria. Participants in the team sport condition are instructed that their team sport choice must follow the basic definition set forth by Eime and colleagues [27], in which at least two participants are active in a necessary collaboration to achieve the objective of the physical activity within a set of rules. These include but are not limited to basketball, soccer, volleyball, dragon boating, and so forth. However, due to the limited nature of team sport seasons, options were somewhat constrained, depending on the time of year. Therefore, to assist with retention and autonomy in terms of choice, the criteria for team sport was slightly adapted and expanded to include registered programs that meet weekly with the same group, such as kickboxing classes, running groups, and bootcamps.

Finally, participants in the individual sport choice condition are provided with a customized handout of our environmental scan of available adult individual sports programs in Greater Victoria (maintained and updated) and asked to choose one of these available options. “Individual sport” is defined as a physical activity performed or completed by an individual with a structured rule set of competition or progression toward an outcome or goal. Individual sports include rowing, running, cycling, swimming, and rock climbing.

Upon allocation, participants are asked to begin their activity as soon as possible (i.e., register for a team sport, choose an individual sport or “personal time” activity, and commence weekly sessions). Participants in both sport conditions are asked to keep track of their attendance in the program. After the initial 6 weeks, participants are sent follow-up questionnaires to complete via email. Contact is made initially with a phone call by the research assistant to answer any questions the participants may have about their experiences over the last 6 weeks. A phone-assisted report of sport participation over the past 6 weeks is also ascertained at this time. A similar protocol is followed at the 3-month mark; however, participants are asked to come to the laboratory to partake in a brief wrap-up interview and an end-of-trial questionnaire (*see* Fig. 1 for schedule of interventions and assessments). To assist study retention, we offer to subsidize the program chosen by participants with a maximum $80 contribution toward registration (or the personal time activity of choice for the control participants) as well as a maximum $25-per-week contribution toward childcare when needed. Participants must complete the study in order to receive the honorarium.

## Results

**Fig. 2:**
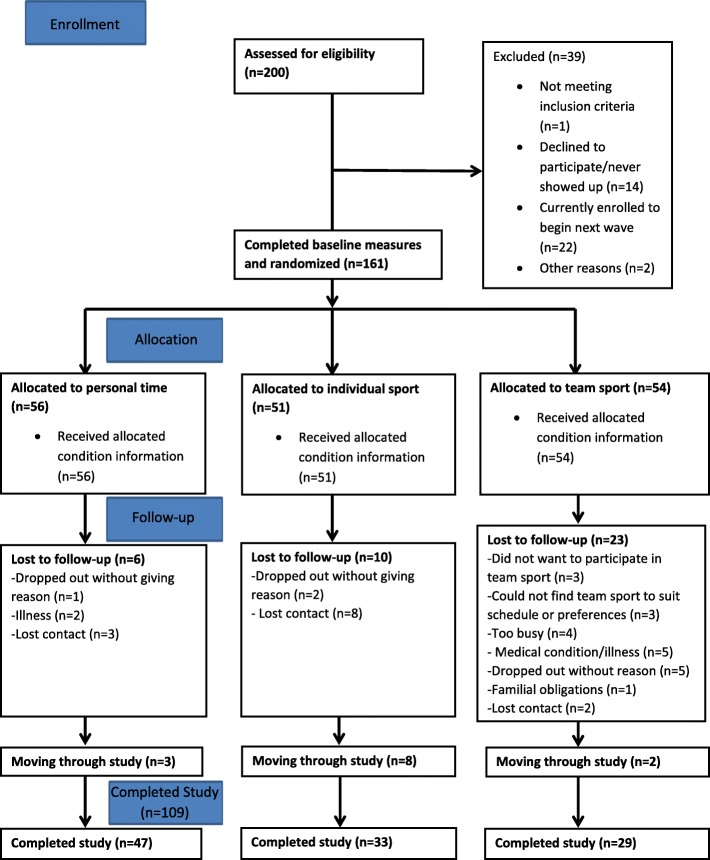
Participant flow diagram

To date, we have obtained ethical approval, registered the trial, and recruited 161 parents from the Greater Victoria region. Ethical approval was received from the University of Victoria Human Research Ethics Board (HREB). Of the 200 parents assessed for eligibility, 22 are currently on the wait-list for the next wave, 161 have completed all of the baseline measures, 127 have completed the 6-week measures, and 109 have completed the 3-month measures concluding the study. *See* Fig. 2 for the participant flowchart.

### Oversight, monitoring, ethics, and dissemination

#### Oversight and monitoring

Because of the relative simplicity of the data collection methods, the research team concluded that a formal data monitoring committee was not required. As an alternative, the project coordinator provides a monthly report of trial progress, including advances toward intended sample size and the quality of the data collected. The principal investigator is responsible for project oversight, will make the final decision to terminate the trial, and will have access to the final trial dataset.

#### Research ethics processes

In the case of any protocol changes, the project coordinator submits the appropriate documentation to the ethics board (HREB at the University of Victoria) to request modifications or amendments. Upon approval, any amendments are communicated to relevant parties, and the trial registry is updated accordingly.

It is anticipated that there will be no harms as a result of participation; nevertheless, participants are provided with contact information for the project coordinator, primary investigator, and HREB in the case of unintended effects or adverse events. All those involved in study implementation are trained to document and report any such events. If such an event occurs, depending on its nature, the safety of all parties involved will be ensured.

#### Dissemination plans

Results of this trial will be widely disseminated through knowledge exchange activities, including presentations at relevant scholarly conferences and publications in scientific journals. All those who have contributed to the design and protocol will be eligible for authorship of subsequent publications. Findings may have important implications for public health and could potentially inform initiatives for adult sport participation. Public access to the participant data set will not be granted. Currently, there are no plans to make the statistical code publicly available (as per our HREB approval). Any relevant publications will be shared with participants who have expressed interest.

## Discussion

This protocol describes the implementation of a randomized controlled trial that investigates the efficacy of two sport participation formats (individual sport and team sport) with regard to key psychosocial outcomes, based on the tenets of the Sport Participation Model, among parents with young children. Research findings will be important to public health because they may help to determine whether sport participation among parents improves psychological health and physical activity participation. Because little empirical evidence of the effect of sport participation among this demographic exists, this information could prove pivotal for policy-level sport interventions geared toward promoting positive psychosocial outcomes for parents.

## Trial status

The protocol (version 2) was updated with the National Library of Medicine on October 10, 2019. Recruitment for this trial started in September 2016 when version 1 of the protocol was initially released. We aim to complete recruitment by December 2021. The study is ongoing as of February 2020, and data analysis will continue through 2022.

## Supplementary information

**Additional file 1.** Completed SPIRIT (2013) Checklist: Details on which page of the manuscript each relevant recommended item is addressed.

**Additional file 2.** Consent form.

## Data Availability

The datasets generated and/or analyzed during the current study are not publicly available as per ethical approval from the HREB at the University of Victoria, which stipulates that the data will not be accessed or analyzed by others.

## Abbreviations

CSEP: Canadian Society for Exercise Physiology
HREB: Human Research Ethics Board at the University of Victoria
PA: Physical activity
SF-12: 12-Item Short Form Health Survey
