# Supplementary material for: Promoting sport participation during early parenthood: a randomized controlled trial protocol
Source: Trials. 2020 Feb 27;21:230. doi: 10.1186/s13063-020-4158-x (PMC7045446; doi:10.1186/s13063-020-4158-x)
Supplement: Supplementary file 2 — Additional file 2. Consent form. [file 13063_2020_4158_MOESM2_ESM.docx]

Additional file 2

**
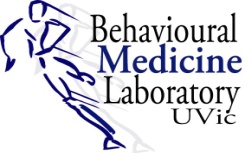

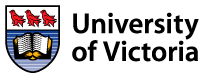
Consent Form**

**“Exploring Parental Satisfaction and happiness through activities”**

You are being asked to take part in a study titled **“Exploring Parental Satisfaction and happiness through activities”** funded by the Social Sciences and Humanities Research Council. We are inviting any parent(s) who currently have at least one child under the age of 13, to participate in this study. This study has been reviewed by the University of Victoria Ethics Committee and has met the rigorous requirements for ethical approval.

Although regular physical activity is linked to higher social functioning, greater vitality and overall psychological well-being, research shows that there is a large decline in sport and physical activity participation among adults as they enter into parenthood. This decline in sport and physical activity could influence overall family functioning and therefore if parents have difficulty maintaining regular activities due to family obligations, children may also be impacted. As a result, the promotion of regular physical activity is paramount in alleviating the potential health implications that may arise for the entire family. We hope that you will help us learn more about factors such as physical activity and sport influence well-being and functioning among parents by participating in the study.

**Purpose of this Project**

In this study, we will be examining whether different activities help to promote parental well-being and overall family functioning. We will be comparing whether there are differences among parents who go on a “night out” versus an “individual sport” versus a “team sport” condition.

**What do I have to do to participate?**

1) First we ask that you sign this consent form.

2) We then will ask you to fill out a baseline questionnaire and will go through the requirements of your particular condition that you are randomized to. The questionnaires include demographics such as ethnicity, education age etc, and we will also ask you about family dynamics, relationship dynamics and quality of life.

3) After six weeks, we will contact you about a second questionnaire via email.

4) At 3 months’ time, we will ask you to come back to the lab to complete a final questionnaire and go through a brief wrap up interview. The interview will occur at our lab at Uvic and will take approximately 30 minutes. We will ask that we record your answers to ensure accuracy.

**Inconvenience, Risks, and Benefits**

There are some risks to engaging in a new sport, activity or physical activity. We will screen you using a “Physical Activity Readiness Questionnaire” that asks you about your health to see if you are able to increase your physical activity. If it is noted that there may be a contraindication to increasing your activity level you will need to obtain doctor’s approval to participate in the study. There also may be risk to participating in sports such as being hit with a ball or falling down. Organized sports have a First Aid kits on site but it is important you are aware that there is always some level of risk involved with sports. If you are uncomfortable with participating in a sport you may withdraw from the study with no questions asked. You may also experience fatigue from any increases in physical activity or sport that you may engage in. Some of the questions on the questionnaires may ask you about sensitive information (such as your well-being, partner dynamics). Your responses are completely confidential and you do not have to answer any questions you do not want. The potential benefits of your participation in this research include increasing yours (and/or your spouses) activity levels as well as compensation for childcare if needed. We will also cover the cost of your sport.

**Anonymity and Confidentiality**

The information from the questionnaires, and interviews will be completely confidential and will be kept anonymous during data analysis and publication of study results. Findings from this study will be presented at scholarly conferences and used for publication in journal articles. All data will be published as group data, and any data kept separate will be identified by ID-number (no name). We will need your contact information in order to provide you with materials and collect materials. However, we can assure you that your confidentiality will be completely protected and only the research team will have access to your contact information. In terms of protecting the confidentiality of your data, the data file and completed questionnaires will be kept in a locked and secure environment on the University of Victoria campus at all times. Only the investigators will have access to the data. The online questionnaire data will be stored on a server for five years and then permanently deleted. Hard copy consent forms will also be kept for five years and then they will be shredded.

**Do I have** **to participate?**

No, your participation in this study is completely voluntary and you have the right to withdraw at any time without consequence. As well, if you choose to withdraw before the three-month follow up, it is up to you whether or not we use that data that we will have collected from you up until that point. It is only through voluntary participation in research projects that we increase our knowledge about issues that are important to health.

If you have any questions or concerns about this study please do not hesitate to contact either Sandy Courtnall (Research Coordinator) or Dr. Ryan Rhodes (Primary Investigator). In addition, you may verify the ethical approval of this study, or raise any concerns you might have, by contacting the Human Research Ethics Office at the University of Victoria (250-472-4545 or [ethics@uvic.ca)](mailto:ethics@uvic.ca)).
**Your signature below indicates that you understand the above conditions of participation in this study, and that you have had the opportunity to have your questions answered by the researchers.**

|  |  | |  | |  |  | | |  |
| --- | --- | --- | --- | --- | --- | --- | --- | --- | --- |
| *Name of Participant* | |  | | *Signature* | | |  | *Date* | |

*****Please sign one copy for the researchers and sign and keep one copy for your records*****

Ryan E. Rhodes, Ph.D., Professor Sandy Courtnall, Project Coordinator

(250) 721-8384 (250) 472-5288

rhodes@uvic.ca bml@uvic.ca
